# Promising effect of *Geranium robertianum* L. leaves and *Aloe vera* gel powder on Aspirin^®^-induced gastric ulcers in Wistar rats: anxiolytic behavioural effect, antioxidant activity, and protective pathways

**DOI:** 10.1007/s10787-023-01205-0

**Published:** 2023-05-15

**Authors:** Basma M. Bawish, Mariem A. Rabab, Safaa T. Gohari, Marwa S. Khattab, Naglaa A. AbdElkader, Samar H. Elsharkawy, Amr M. Ageez, Manal M. Zaki, Shaimaa Kamel, Eman M. Ismail

**Affiliations:** 1 Department of Veterinary Hygiene and Management, Faculty of Veterinary Medicine, Cairo University, Giza, 12211 Egypt; 2 Faculty of Biotechnology, October University for Modern Sciences and Arts (MSA), 6th October City, 12573 Egypt; 3 Department of Nutrition, Food Science and Home Economics, Faculty of Specific Education, Ain Shams University, Ain Shams, 11566 Egypt; 4 Department of Pathology, Faculty of Veterinary Medicine, Cairo University, Giza, 12211 Egypt; 5 Department of Surgery, Anesthesiology, and Radiology, Faculty of Veterinary Medicine, Cairo University, Giza, 12211 Egypt; 6 Department of Biochemistry and Molecular Biology, Faculty of Veterinary Medicine, Cairo University, Giza, 12211 Egypt

**Keywords:** Polyphenols, Chemical composition, Ultrasonography, Anxiety-like behaviour, TNF-α, Oxidative stress biomarkers

## Abstract

**Background:**

Many drugs have been restricted in the treatment of gastric ulcers (GU). So, herbal medicines are now in great demand for their better cultural acceptability, compatibility, and minimal side effects. Therefore, our study aimed to assess the protective efficacy of *Aloe vera* gel and *Geranium robertianum* extracts against Aspirin®-induced GU in Wistar rats.

**Methods:**

Antioxidant activity and chemical composition of both herbs were analysed. Then, we divided forty female Wistar rats into five groups: a negative control group, a positive control group of Aspirin®-induced GU, and pretreated groups with *Aloe Vera*, geranium, and Famotidine (reference drug). The locomotor disability, anxiety-like behaviour, and ultrasonography were assessed. Ultimately, scarification of animals to determine gastric juice pH and ulcer index. Then the collection of stomach and liver for histopathological and immunohistochemical examinations, besides tracing the oxidative stress biomarkers and related genes.

**Results:**

High content of polyphenols was revealed in both extracts. The pretreatment with *Aloe vera* gel and geranium showed significant antioxidant activities with free radical scavenging and ferric-reducing power (FRAP). Moreover, they improved the stomach architecture and alleviated anxiety-like behaviour and motor deficits. They significantly reduced the expression of proinflammatory cytokine (TNF-α), inflammatory, and oxidative stress genes (NF-KB, HO-1, Nrf-2) while increasing the Keap-1 in gastric mucosa.

**Conclusion:**

Data presented a significant protective effect of *Aloe vera* gel and geranium against Aspirin®-induced GU; they reduced gastric mucosal injury with potential anxiolytic effects through their anti-inflammatory and antioxidant properties. Therefore, they may be considered promising agents for preventing or treating gastric ulceration.

## Introduction

Gastric ulcer (GU) is one of the most pervasive and frequent gastrointestinal disorders, with rising morbidity and mortality rates worldwide (Xie et al. 2017; Chow and Sung 2009). Gastritis and GU are acid-induced gastric diseases caused by pepsin, gastric acid secretion, and a decrease in protective factors characterised by mucosal damage (Ramakrishnan and Salinas 2007). Patients with digestive troubles were discovered to have a high frequency of neuropsychiatric illnesses such as depression and anxiety-like behaviour (Bercik et al. 2010; Goodwin et al. 2013). As gastrointestinal inflammation is highly related to the response of the Hypothalamic Pituitary Adrenal Axis (HPA) to stress, which in turn stimulates the expression of corticotropin-releasing factor (CRF) in the hypothalamus's paraventricular nucleus (PVN) and translated lately to the increase in anxiety-like behaviour (Liu et al. 2011). Remarkably, multifactorial determinants are implicated in the development of GU, such as smoking, alcohol, helicobacter infection, psychological stress, and excessive use of nonsteroidal anti-inflammatory drugs (NSAIDs) (Awaad et al. 2013). The imbalance between these harmful factors in the gastric lumen and protective mechanisms in the gastroduodenal mucosa led to severe gastric irritation and GI symptoms ranging from uncomplicated dyspepsia to potentially fatal GI bleeding and perforations (Almuzafar 2018; Yap and Goh 2015).

Considering NSAIDs, GU has become an upsetting health problem associated with the repeated use of these medicines such as aspirin, ibuprofen, and naproxen. Where multiple mechanisms are involved: inhibiting Cyclooxygenase-2 enzyme (COX-2), which in turn inhibits the formation of prostaglandins, a cell-protective substance found in the gastrointestinal tract (GIT) (Robert et al. 1977), increases the release of reactive oxygen species (ROS), lipid peroxidation, infiltration of neutrophils, and imbalance of proinflammatory mediators (Adhikary et al. 2011). The high levels of cytokines release, like TNF-a (Tumor necrosis factor-a), IL-6 (interleukin-6), and IL-10 (interleukin-10) caused by aspirin ingestion are critical in the acute phase of inflammation and severity of gastritis (Raghavendran et al. 2011). H2 receptor blockers or proton pump inhibitors are the principal therapy for relieving GU symptoms. They regulate stomach acid production and promote ulcer healing (Chan et al. 2017). However, these agents are restricted due to their side effects: tolerance development, relapse incidences, and drug interactions (Srinivas et al. 2013). As a result, it has become critical to create more effective candidates for treating stomach ulcers.

The World Health Organization (WHO) estimated that the first line of treatment for 75–80% of the world's population relies on conventional medicinal preparations due to the safety concerns of synthetic medicines (Hossain et al. 2014; Urbi and Zainuddin 2015; Hossain and Urbi 2016). Furthermore, evidence of the relevance of plants for various diseases in historical books caught the interest of many researchers who focused on analysing the scientific validity of traditional claims (Urbi et al. 2014; Hossain 2016; Hossain et al. 2021). Herbal medications treat GU by stimulating mucous cell growth, reducing oxidative stress, and inhibiting gastric acid secretion (Bi et al. 2014). Many herbs constitute polyphenols, a group of compounds containing phenolic hydroxyl attached to ring structures, which function as antioxidants. These compounds are reducing agents, singlet oxygen quenchers, and hydrogen-donating antioxidants (Suresh et al. 2012). *G. robertianum* L. (Geraniaceae family) is known as Herb Robert or Red Robin and is native to Europe, Asia, North America, and North Africa (Lis-Balchin 2002). It has been used in folk medicine for a long time in several countries for many therapeutic purposes, as it has several phenolic, tannin, and flavonoid compounds (Paun et al. 2012; Neagu et al. 2013; Santos et al. 2013; Igwenyi and Elekwa 2014). This medicinal plant is popular due to the remedy of several digestive system disorders and its anti-inflammatory, antiallergic, antidiarrhoeic, anti-diabetic, antibacterial, anti-cancer, antihepatotoxic, diuretic, hemostatic, and tonic properties (Graça et al. 2016). In addition, stress, anxiety, and depression are currently treated with geranium-based herbal medicines (Tabari et al. 2018). The essential chemicals discovered in *G. robertianum* L. have antiulcer properties, including anti-acid secretion, suppression of pepsin, and increased stomach mucus and bicarbonate (Serafim et al. 2020). *Aloe vera* (Aloe barbadensis Miller), a Liliaceae family, is a perennial, xerophytic, succulent, shrubby or arborescent plant with a pea-green colour, primarily found in dry areas of Africa, Asia, Europe, and America (Surjushe et al. 2008). It contains over 75 compounds, including amino acids, sugars, fatty acids, vitamins, minerals, salicylic acid, lignin, and saponins (Dureja et al. 2005). Multiple biological properties of *Aloe vera* have been described, including wound healing, anti-cancer, immunological modulation, antioxidant, antimicrobial, and gastroprotective activities (Takzare et al. 2009; Im et al. 2010; Borra et al. 2011; Fani and Kohanteb 2012). *Aloe vera's* antiulcer properties have been linked to their anti-inflammatory characteristics, healing, mucus stimulatory effects, and stomach secretion modulation (Eamlamnam et al. 2006). A previous study indicated that oral consumption of *Aloe vera* gel might have sedative and anxiolytic effects in rodents (Embark and Abdalla 2019). Additionally, reducing depression-like behaviour (Halder et al. 2013) and improves motor behavioural activities in diabetic mice (Parihar et al. 2004).

To our knowledge, no previous study has investigated the protective activities of *G. robertianum* L. and *Aloe vera* against gastric ulcers. Accordingly, our research aimed to assess the potential protective efficacy of both herbs against Aspirin®-induced GU in rats by assessing the essential mechanisms involved in such protection. Moreover, the motor activity and anxiety-like behaviour associated with gastric ulceration are assessed and determined.

## Materials and methods

### Plant preparation and analysis

Two kilograms of *G. robertianum* L. and *Aloe vera* (L.) Burm F*.* (Liliaceae) leaves were obtained from the Orman-botanic garden in Giza, Egypt. Samples were identified and authenticated at Cairo University Research Park (CURP). At the same time, Famotidine and Aspirin® (acetylsalicylic acid, NSAID) were obtained from El-Gomhoreya Co., Cairo, Egypt. The inner gel of Aloe *Vera* leaves was collected using a clean, sharp knife after carefully washing and cutting them into pieces; then, it was subjected to hot air drying overnight at 55 ºC. Subsequently, the administration dose was adjusted to 50 mg/mL after the dried gel powder was suspended in distilled water and stored in tightly sealed dark containers at − 20 °C for later use (Misawa et al. 2012). Additionally, the preparation of leaf aqueous extract of *G. robertianum* L. was done as reported by Amabeoku et al. (2007), where one kg of leaves was washed and dried at 35 °C for 4 days. Then, the dried leaves were ground to a fine powder (850 m) to obtain a yield of 185.3 g; then, 80 g of fine powder was refluxed in 1 L of boiling water, allowed to cool, and filtered. Ultimately, the filtrate was frozen at − 80 °C and freeze-dried for 120 h.

### Analytical methods

The proximate composition of protein, fat, ash, fibre and moisture content of both *G. robertianum* L. and *Aloe vera* gel powder was determined according to AOAC (2015), and then the carbohydrate content was calculated by difference (Fernandes et al. 2015).

$$ {\text{Total carbohydrates}} = 100 - \left( {{\text{moisture}} + {\text{protein}} + {\text{fat}} + {\text{ash}}} \right) $$

Then plant extracts were prepared to determine polyphenols acc. to AOAC (2015) with some modifications. Where 10 g of *G. robertianum* L. *leaves* and *Aloe vera* gel powder was extracted in 100 mL of water using the ultrasonic device (200 W, 59 kHz, Shanghai Kudos Sonication Machine Company Ltd., China) for 60 min at room temperature, then the extracts were centrifuged for 15 min at 4000 rpm. The Folin–Ciocalteu assay was followed to characterise polyphenols in the two plant extracts, adapted from Ramful et al. (2011). The analysis was performed using liquid chromatography-electrospray ionisation–tandem mass spectrometry (LC–ESI–MS/MS).

### Antioxidant activities: free radical scavenging activity calculation/ferric reducing power (FRAP) determination

The effect of the two plant extracts on the free radical, 1,1-diphenyl-2-picrylhydrazyl (DPPH), was calculated using the procedure described by Aboelsoued et al. (2019). A spectrophotometer was used to measure the absorbance of samples at 517 nm, and ethanol was used as a control. We calculated DPPH scavenging capacity using the following equation:

$$ {\text{Scavenging activity}}\, \left( \% \right) = \left( {{\text{Ac}} - {\text{As}}} \right)/{\text{Ac}} \times 100. $$

Ac and As are the absorbances at 517 nm of the control and sample, respectively.

The ferric reducing power (FRAP) was determined by relying on phenolics' ability to reduce Fe^3+^ to Fe^2+^. The FRAP reagent was made by combining 0.1 M acetate buffer (pH 3.6), 10 mM TPTZ, and 20 mM ferric chloride (10:01:01, v/v/v). To 150 L of reagent, 20 µL of previously diluted extract were added. A Microplate spectrophotometer was used to measure absorbance at 593 nm. The analysis was carried out in triplicate, with an aqueous Trolox solution serving as the standard, and the results were expressed as lmolesTrolox equivalents/100 g of the sample (Barros et al. 2012).

### Experimental study for induction of gastric ulcer in rats

Forty female Wistar Albino rats (150 ± 20 g) were obtained from Animal Breeding Laboratory, VACSERA, Helwan, Egypt. All rats were housed at the Laboratory Animal Unit, Faculty of Veterinary Medicine, Cairo University, Egypt, and kept under standard conditions at a temperature of (25 ± 1 °C), relative humidity (50%) with a 12-h light/dark cycle. All rats were maintained on a standard diet and water ad libitum. [14% casein, 10% sucrose, 4% corn oil, 5% fibre (cellulose), 3.5% mineral mixture, 1% vitamin mixture, 0.25% choline chloride, 0.3% D-L methionine, and 61.95% corn starch] (Reeves et al. 1993). All the experimental procedures were carried out acc. to the guidelines of the Veterinary Institutional Animal Care and Use Committee (VET-IACUC), Faculty of Veterinary medicine, Cairo University, Egypt (Ethical reference No: Vet CU 2305 2022476). After 1 week of acclimation, the rats were equally and randomly divided into five groups (eight animals each). Group (1), the negative control group, received (2 mL/kg BW) distilled water/day/animal orally by epi-gastric tube for 10 days. Group (2), Aspirin®-induced gastric ulcer group (positive control group) received (2 mL/kg BW) distilled water orally. Group (3) pretreated orally with 300 mg/kg *Aloe Vera* suspension gel (Bahrami et al. 2020). Group (4) pretreated orally with 400 mg/kg leaf aqueous extract of *G. robertianum* L. (Necib and Zekri 2020). While group (5), the reference drug group, received orally (50 mg/kg BW) Famotidine suspended in distilled water (Alazzouni et al. 2021). On the 10th day, all rats fasted for 24 h and received a single oral dose of Aspirin® at a dose of 500 mg/kg BW suspended in distilled water for the induction of acute gastric ulcer except for rats of the control group (1) (Mahmoud and Abd El-Ghffar 2019). One hour after administration of all treatments, all rats were subjected to behavioural examinations to assess locomotion and anxiety-like behaviour, then ultrasonographic examination to determine the thickness of the gastric wall. At the end of the experiment, the animals were killed to collect stomach and liver samples for further analysis to determine the potential and essential mechanisms involved in treating gastric ulceration.

### Behavioural examinations

#### Open-field test (OFT)

They were used to compare the effects of *Aloe vera, Geranium,* Famotidine, and Aspirin® on locomotor and anxiety-like behaviour in female Wistar rats. So, OF test was performed inside a square wooden box (70 × 70 × 35 cm height) with a floor divided by black lines into 16 squares of equal size. One hour after administration of all treatments, rats were gently and alternately placed in one corner of the open field, and the behaviour of each animal was videotaped by a video camera positioned above the centre of the maze for 3 min. During this, the following behaviours were scored: the ambulation frequency (the number of squares crossed by the rat), rearing frequency (the number of times the animals stood on their hind legs), and freezing duration (total motionless time). The ambulation and rearing frequencies were taken as indices of locomotor behaviour, while freezing duration was taken as an index of anxiety-like behaviour. The box was wiped out with a cloth soaked in 70% ethyl alcohol and allowed to dry between tests to avoid olfactory cues (Gould et al. 2009).

#### Elevated plus maze test (EPM)

The EPM test was conducted with four crossed arms, two open arms with no side walls (50 × 10 cm), and two closed arms (50 × 10 × 30 cm). The maze was raised 60 cm above the ground. The rat was placed in the maze's centre and allowed to explore the entire maze for 5 min. The number of entries in open and closed arms was counted, and the time spent by the animal in the open and closed arms during 5 min period was recorded via a video camera installed above the centre of the arena. After each trial, the arena was cleaned and sterilised with 70% ethyl alcohol and allowed to dry (Walf and Frye 2007).

### Ultrasonographic examination

It has been performed in the Department of Surgery, Anesthesiology, and Radiology, Faculty of Veterinary Medicine, Cairo University. In all groups, the ventral portion of the abdomen was carefully clipped at 1 cm cranial to the xiphoid cartilage in the caudal-most region of the pubis. Following the same ventral approach, a complete ultrasonographic examination was performed with the animal in dorsal recumbency, using contact jell on the abdomen of the examined rat, using HITACHI ALOKA Ultrasonic Diagnostic Imaging System with liner transducers of multi-frequency 5–13 MHz.

### Assessment of gastric mucosal damage (Ulcer Index, UI)

After the killing of rats, their stomachs were wrapped around their pyloric and cardiac sphincters and then injected with 3 mL of distilled water. A sterile tube was then used to collect the gastric juice. The ulcer index was determined using a magnifying lens, as stated by Bandyopadhyay et al. (2004). The sum of the area of the lesions for each stomach was used to calculate the UA (Ulcer Area) (Robert et al. 1984). The ulcer index was calculated using the following formula:

$$ {\text{UI}}\% = \left[ {\left( {{\text{UA of C}} - {\text{UA of T}}} \right) \div {\text{UA of C}}} \right] \times 100\% . $$

UI is the ulcer inhibition, T is the treatment, and C is the negative control. The gastric juice from each animal was centrifuged at 3000 rpm for 10 min to remove any solid debris. Gastric juice volume was measured with a graduated cylinder and expressed in millilitres (mL) (Moore 1968). The supernatant's pH was then calculated following Debnath et al. (1974).

### Determination of malondialdehyde (MDA) and reduced glutathione (GSH)

The stomach and liver samples were weighed to determine oxidative stress biomarkers. Then, they were homogenised with phosphate buffer saline and centrifuged at 12,000 rpm for 10 min to prepare 10% tissue homogenate. MDA was determined in the tissue homogenate by using a Bio-diagnostics kit. GSH was determined in the tissue homogenate, acc. to Ellman (1959).

### Gene expression analysis

For determination of the transcript levels of NF-kB, COX-2, HO-1, Keap-1, and Nrf-2 genes, approximately 100 mg of the stomach and liver samples from each group were utilised to extract total RNA utilising ABT Total RNA Mini Extraction Kit, Applied Biotechnology (Cat. # ABT002). The quantity and quality of RNA were determined by Nanodrop Technology (Kamel et al. 2018a). The cDNA was synthesised using ABT H-minus cDNA synthesis kit, Applied Biotechnology. Quantitative assessment of cDNA amplification for the studied genes was done by quantitative Real-Time PCR (qRT-PCR) utilising ABT 2X SYBR GREEN Master Mix, Applied Biotechnology. Internal control was achieved using the GAPDH gene (Hassanen et al. 2023). The primers used in qRT-PCR were designed by the primer designing tool acc. to the national centre for biotechnology information (NCBI) https://www.ncbi.nlm.nih.gov/tools/primer-blast/index.cgi?LINK_LOC=BlastHome and were demonstrated in Table 1. Each qRT-PCR was done in triplicates (Kamel et al. 2018b). The equation of 2^−ΔΔCT^ was utilised to calculate the expression levels of different genes (Kasas et al. 2022).

**Table 1:** Primers sequences of qRT-PCR

Gene symbol	Gene description	Accession number	Primer sequence
NF-Kb	Nuclear factor kappa B	NM_001276711.1	F: 5′‐CACTGTCAACAGATGGCCC‐3′
			R: 5′‐GTCTGTGAGTTGCCGGTCTC‐3′
COX-2	Cyclooxygenase-2	NM_017232.3	F: 5′‐AAGCCTCGTCCAGATGCTA-3′
			R: 5′‐ATGGTGGCTGTCTTGGTAGG-3′
HO-1	Heme oxygenase 1	NM_012580.2	F: 5′-AGCGAAACAAGCAGAACCCA-3′
			R: 5′-ACCTCGTGGAGACGCTTTAC‐3′
Keap-1	Kelch-like ECH-associated protein 1	NM_057152.2	F: 5′-ATGTGATGAACGGGGCAGTC‐3′
			F: 5′-AAGAACTCCTCCTCCCCGAA‐3′
Nrf-2	Nuclear factor, erythroid 2-like 2	NM_031789.2	F: 5′‐ TGTAGATGACCATGAGTCGC‐3′
			R: 5′‐TCCTGCCAAACTTGCTCCAT‐3′
GAPDH	Glyceraldehyde3-phosphate dehydrogenase	NM_017008.4	F:—5′-ACCACAGTCCATGCCATCAC-3′
			R:—5′-TCCACCACCCTGTTGCTGTA-3′

### Histopathological and immunohistochemical examinations

Stomach and liver specimens from different groups were fixed in 10% neutral buffered formalin. Tissues were then processed by xylene and ascending grades of ethanol, embedded in paraffin, and sectioned by microtome into 3–4 µm thick sections. Tissue sections were stained by hematoxylin and eosin stain and periodic acid Schiff (PAS) stain. Light microscopy with a digital camera examined the stained tissue sections. Tumour necrosis factor alpha (TNF-α) was detected using mouse anti-TNF-α (Santa Cruz Biotechnology, Santa Cruz, California, USA) in gastric tissue after antigen retrieval by sodium citrate as primary antibodies. The avidin–biotin–peroxidase method was then applied according to the manufacturer's protocol, and diaminobenzidine (DAB) was used to develop the colour. Mayer's hematoxylin was used as a counterstain. The negative control slides did not utilise primary antibodies.

### Statistical analysis

Shapiro–Wilk and Levene's tests evaluated variances' normality and homogeneity of variances. Then, the data were statistically subjected to analysis of variance (ANOVA) using PASW Statistics, Version 24.0. software (SPSS Inc., Armonk, NY, USA). Tukey’s test post hoc analysis was used to determine the significant difference between means. Data were represented as Mean ± SEM. Values were considered significant at *P* < 0.05, otherwise were considered non-significant. GraphPad Prism version 6.00 were used to create the graphs (GraphPad Software, San Diego, CA, USA).

## Results

### Proximate plant chemical composition

Table 2 lists the percentages of chemical compositions for each extract. Findings showed that ash, protein, and fat content in *G. robertianum* L. leaves were higher (19.44, 2.11, and 1.08%) than in *Aloe vera* gel powder (0.58, 1.51, and 0.89%), respectively. *Aloe vera* gel powder was distinguished by having a higher percentage of carbohydrates and moisture (90.16 and 6.87%) than *G. robertianum* L. leaves (71.83 and 5.54%, respectively).

**Table 2:** *G. robertianum* L. leaves and powdered *Aloe vera* gel's approximate chemical composition

Constituent (%)/samples	*G. robertianum* leaves	*Aloe vera* gel powder
Moisture	5.54	6.87
Protein	2.11	1.50
Fat	1.086	0.89
Ash	19.44	0.58
Carbohydrate	71.83	90.16

### Polyphenols and antioxidant properties

High concentrations of polyphenols were determined in both herbs (Table 3). However, the polyphenols content of *G. robertianum* L. and *Aloe vera* gel powder varied significantly (*p*≤0.05), ranging from 194.962.15 to 10.14.44 mg gallic acid/g for *G. robertianum* L. and *Aloe vera* gel powder, respectively. Significant differences (*P* ≤ 0.05) between *G. robertianum* L. and *Aloe vera* gel powder in the antioxidant activities are shown in Table 3. *G. robertianum* L. demonstrated greater radical scavenging capacities for DPPH and TPTZ (78.29 ± 4.59% and 152.45 ± 1.84 µgTroloxeq/g sample, respectively) than *Aloe vera* gel powder (11.621.31% and 51.042.20 gTroloxeq/g sample, respectively).

**Table 3:** Polyphenols and the antioxidant activities of *G. robertianum* L. leaves and *Aloe vera* gel powder

Samples/compounds	Polyphenols (mg GAE/g)	DPPH%	TPTZ (µgTroloxeq/g sample)
*G. robertianum* leaves	194.96 ± 1.24^a^	78.29 ± 2.65^a^	152.45 ± 1.06^a^
*Aloe vera* gel powder	10.14 ± 0.25^b^	11.62 ± 0.76^b^	51.04 ± 1.27^b^

^a,b^Mean values with different superscripts in the same column indicate significant differences (Tukey's test; *P* ≤ 0.05), and data were presented as means ± SEM

### Characterisation of polyphenols by LC/MS/MS

Following LC/MS/MS analysis, 21 compounds were detected and identified (Table 4). Chlorogenic acid (443.84 ug/g), rutin (230.84 ug/g), gallic acid (115.65 ug/g), naringenin (56.02 ug/g), dihydroxybenzoic acid (29.95 ug/g), myricetin (18.44 ug/g), quercetin (18.16 ug/g), vanillin (16.19 ug/g) and catechin (13.66 ug/g) were the most abundant compounds in *G. robertianum* leaves. In comparison, the most prevalent substances in *Aloe vera* gel powder were dihydroxybenzoic acid (69.46237 ug/g), coumaric acid (3.454931 ug/g), and naringenin (3.392882 ug/g).

**Table 4:** The LC/MS/MS polyphenols characterisation of powdered *Aloe vera* gel and *G. robertianum L.* leaves

Compounds/samples	*Aloe vera* gel powder (ug/g)	*G. robertianum* L. leaves (ug/g)
Chlorogenic acid	0.998635	443.84
Gallic acid	0.794058	146.87
Daidzein	0.012899	0.08
Rutin	0.039435	230.84
Vanillin	0	16.19
Coumaric acid	3.454931	7.08
Naringenin	3.392882	56.02
Caffeic acid	0	3.11
Ellagic acid	1.816514	115.65
Querectin	0.245858	18.16
Dihydroxybenzoic acid	69.46237	29.95
Ferulic acid	0	5.46
Syringic acid	0	7.58
Methyl gallate	0	0.71
Myricetin	0	18.44
Luteolin	0.34652	4.29
Hesperetin	0	0.00
Kaempferol	0	1.24
Apigenin	0	0.00
Catechin	0	13.66
Cinnamic acid	0	0.00

### Behavioural tests

**Fig. 1:**
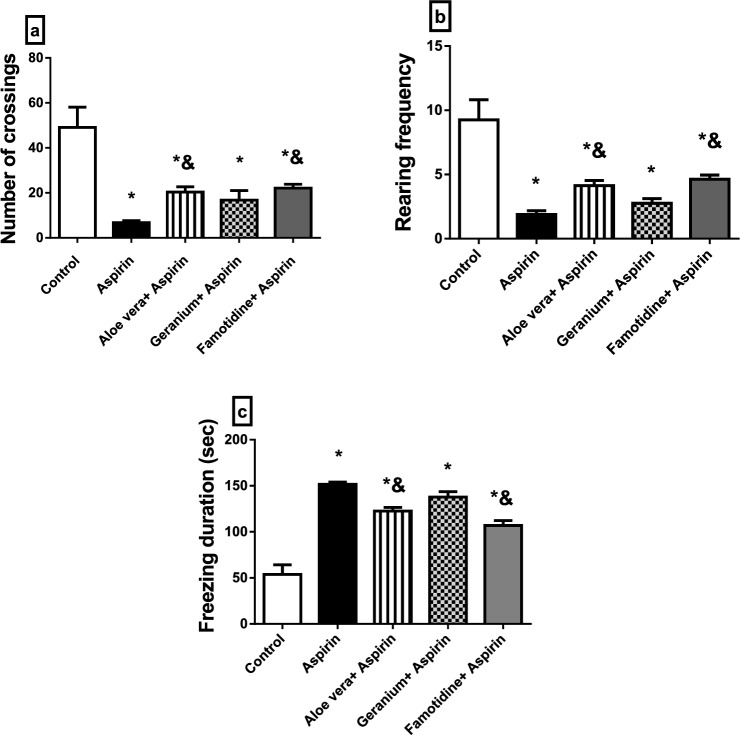
The effect of *Aloe vera* or geranium on locomotion and anxiety-like behaviour in rats with Aspirin®-induced gastric ulcers in an open field test (OFT). **a** The number of crossings, **b** rearing frequency, **c** freezing duration. Values are represented as mean ± standard error (SEM) (*n* = 8). * Significantly different from the control group, & significantly different from the Aspirin group, *P* < 0.05

**Fig. 2:**
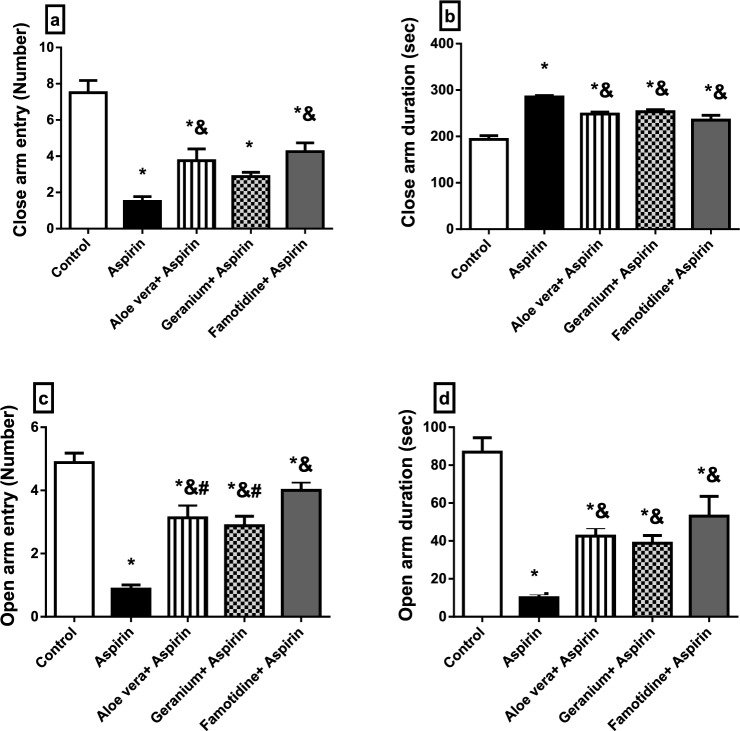
The effect of *aloe vera* or geranium on the anxiety-like behaviour in rats with Aspirin®-induced gastric ulcers in Elevated plus maze test (EPM). **a** Close arm entry, **b** close arm duration, **c** open arm entry, **d** open arm duration. Values are represented as mean ± standard error (SEM) (*n* = 8). * Significantly different from the control group, & significantly different from the Aspirin group, # significantly different from the Famotidine + Aspirin group, *P* < 0.05

In the examined rat groups, there was evidence of pain-related and inflammatory GIT problems linked to anxiety symptoms and motor deficits or impairment. According to our research, rats in the Aspirin®-treated group displayed increased anxiety-like behaviour and a substantial increase in freezing time (*P* ≤ 0*.*05). However, ambulation and rearing frequency in OFT significantly decreased (*P* ≤ 0*.*05) compared to the control group, indicating reduced motor activity and exploratory behaviour (Fig. 1a–c). According to our findings, the anxiogenic impact of Aspirin® administration was observed by a substantial decrease in the frequency of closed-arm entry, open-arm entry, and time spent in the open arm, while it increased time spent in the closed arm significantly (*P* ≤ 0*.*05) when compared to the control group (Fig. 2a–d). Compared to Aspirin®-treated rats, pretreatment with either Famotidine, *Aloe vera*, or geranium reduced anxiety-like behaviour in rats as evidenced by an increase (*P* ≤ 0.05) in ambulation and rearing frequency and a decrease in freezing time which suggests the ameliorative effect of these supplements on anxious rats (Fig. 1a–c). Furthermore, in EPM, the frequency of closed-arm entry, open-arm entry, and time spent in the open arm increased significantly (*p*≤0.05); however, time spent in the closed-arm decreased significantly (*P* ≤ 0*.*05) when compared to the Aspirin® group (Fig. 2a–d).

**Fig. 3:**
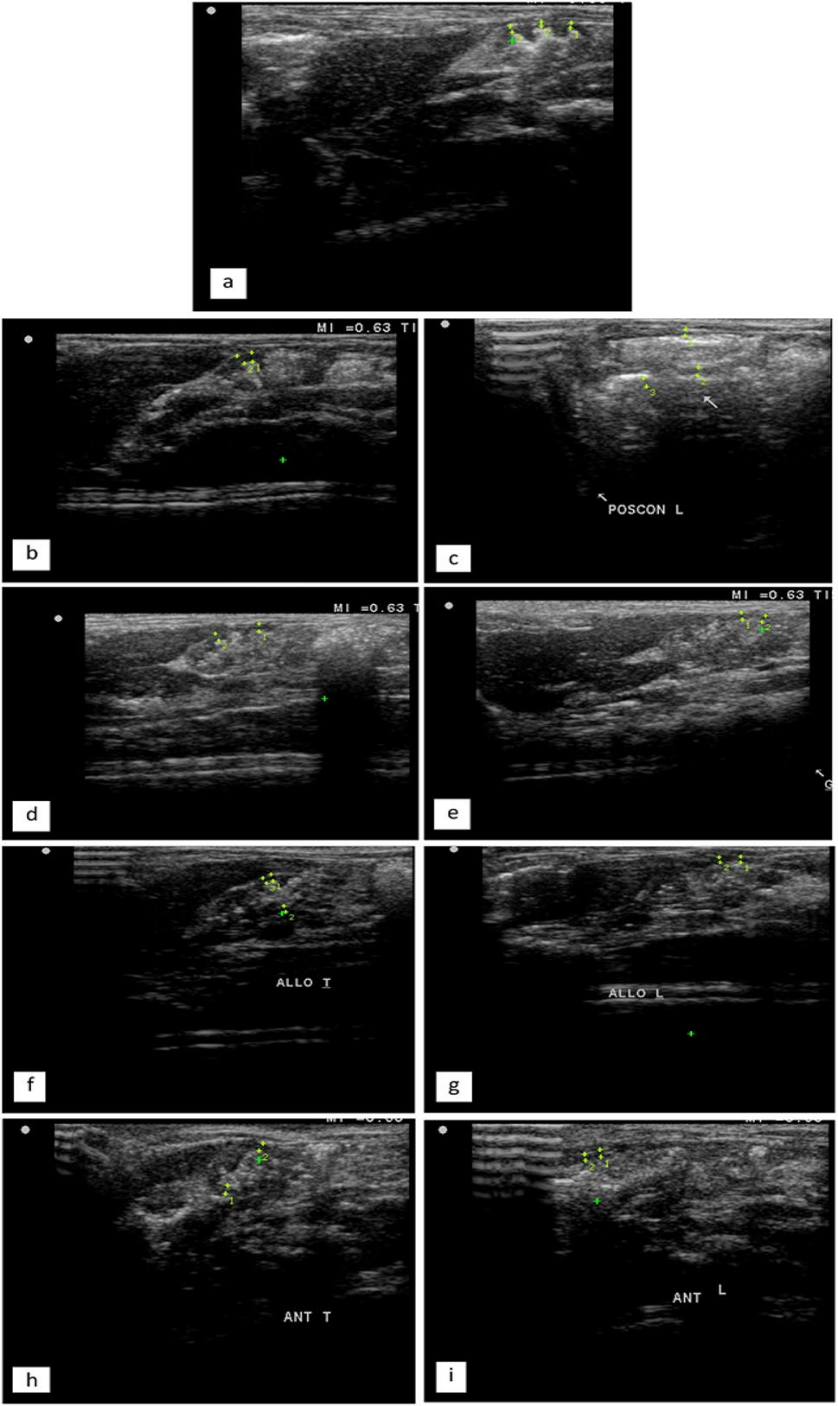
**a** Transverse ultrasonographic scan of the stomach showed standard gastric wall thickness (0.6 mm and 0.5 mm) in the negative control group. **b**, **c** Transverse and longitudinal ultrasonographic scans of the stomach show thickened gastric walls (1.2 mm and 1.1 mm, respectively) in the positive control group. **d**, **e** Transverse and longitudinal ultrasonography scans of the stomach showed standard gastric wall thickness (1 mm and 0.9 mm, respectively) in the Germanium group. **f**, **g** Transverse and longitudinal ultrasonography scans of the stomach showed standard gastric wall thickness (0.7 mm, 0.8 mm, and 0.9 mm) in the *Aloe vera* group. **h**, **i** Transverse (A) and longitudinal (B) ultrasonographic scans of the stomach showed standard gastric wall thickness (0.9 mm and 0.7 mm, respectively) in the Famotidine group

### Ultrasonographic examination

The negative control group's stomach ultrasonographic scan revealed an average gastric wall thickness of 0.5–0.6 mm (Fig. 3a), while the positive control group's ultrasonographic scan showed a thickened gastric wall of 1.2 mm and 1.1 mm (Fig. 3b, c). The Germanium group among the pretreatment groups had an average stomach wall thickness of 1 mm and 0.9 mm, respectively, according to an ultrasonographic scan (Fig. 3d, e). The *Aloe vera* group's ultrasonographic scan also revealed average stomach wall thicknesses of 0.7 mm, 0.8 mm, and 0.9 mm (Fig. 3f, g). The Famotidine group's ultrasonographic scan similarly demonstrated an average stomach wall thickness of 0.9 mm and 0.7 mm (Fig. 3h, i). In the recent study, the standard gastric wall thickness was within the normal range using an ultrasonographic scan for the negative control group, the Germanium group, the *Aloe vera* group, and the Famotidine group (1.07–1.09 mm). In contrast, the positive control group's ultrasonographic scan revealed a thicker stomach wall of 1.2 mm and 1.1 mm compared to the negative control group and the normal range of ultrasonographic readings for the rat gastric wall (mm). Rats' gastric walls typically measure between 1.09 and 0.20 mm in the saccus cecus and between 1.07 and 0.25 mm in the fundus wall (Banzato et al. 2014).

### Volume and pH of gastric juice

When compared to the control animals (−), Aspirin® induction could considerably (*p*≤0.05) increase the gastric juice volume and decrease the gastric content pH in the ulcer group (+). (Table 5). When compared to the ulcer control group (+) and the reference control group (Famotidine group)., the pretreated groups with aqueous extracts of geranium leaves (400 mg/kg) and *Aloe vera* gel Powder (300 mg/kg) have antisecretory activity as evidenced by a decrease in the volume of gastric juice and an increase in the pH of the gastric contents.

**Table 5:** Effect of aqueous extract of *G. robertianum* L., *Aloe vera* gel powder, and Famotidine on the volume and pH of gastric juice induced by Aspirin®

Groups	Volume (mL)	pH
Control (−)	0.28 ± 0.004^b^	3.20 ± 0.00^a^
Control (+)	1.12 ± 0.008^c^	1.30 ± 0.00^c^
*G. robertianum* L. (400 mg/kg)	0.45 ± 0.008^a^	3.31 ± 0.25^a^
*Aloe vera* gel (300 mg/kg)	0.46 ± 0.01^a^	3.22 ± 0.00^a^
Famotidine (50 mg/kg)	0.43 ± 0.04^b^	2.67 ± 0.00^b^

^a,b,c^Mean values with different superscripts in the same column indicate significant differences (Tukey's test; *P* ≤ 0.05), and data were presented as means ± SEM

### Ulcer index assessment

In Table 6, compared to the control group, Aspirin® administration resulted in a remarkably high ulcer index (8.71 ± 0.32). The experimental rats were protected significantly (*p*≤0.05) against Aspirin®-induced gastric ulcers by pretreatment with *G. robertianum* L., *Aloe vera* gel powder, and Famotidine. Aqueous *G. robertianum* L. leaf extract (400 mg/kg) decreased the ulcer index to 1.00 ± 0.08, indicating an 88.5% success rate in preventing them. The ulcer index was also decreased to 1.37 ± 0.19 by *Aloe vera* gel powder (300 mg/kg), indicating a corresponding 84.2% prevention. Famotidine (50 mg/kg) decreased the ulcer index to 2.70 ± 0.08, demonstrating a 69.0% reduction in the risk of stomach mucosal damage.

**Table 6:** Effect of aqueous extracts of *G. robertianum* L., *Aloe vera* gel powder, and Famotidine on gastric lesion surface induced by Aspirin®

Groups	Gastric mucosal injury area (mm^2^)	Protection (%)
Control (−)	0 ± 0.00^e^	–
Control (+)	8.71 ± 0.32^a^	–
*G. robertianum* (400 mg/kg)	1.00 ± 0.08^d^	88.5
*Aloe vera* gel (300 mg/kg)	1.37 ± 0.19^c^	84.2
Famotidine (50 mg/kg)	2.70 ± 0.08^b^	69.0

^a,b,c,d,e^Mean values with different superscripts in the same column indicate significant differences (Tukey's test; *P* ≤ 0.05), and data were presented as means ± SEM

### Biochemical analysis

#### Oxidative stress

**Fig. 4:**
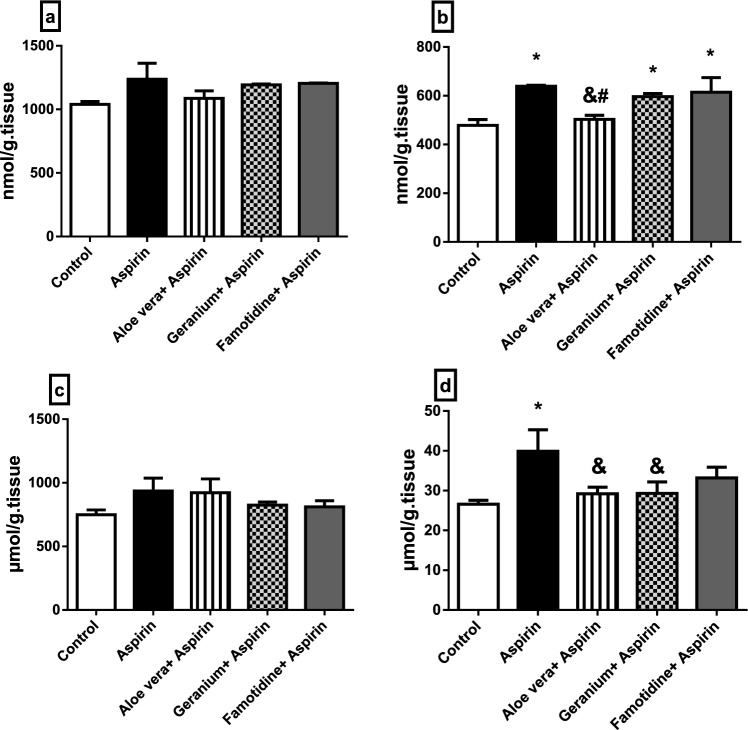
**a**–**d** Oxidative stress biomarkers in the stomach and liver of rats: **a** MDA in the liver; **b** MDA in the stomach; **c** GSH in the liver; **d** GSH in the stomach. Data are represented as mean ± SEM. * Significantly different from the control group, & significantly different from the Aspirin group, # significantly different from the Famotidine + Aspirin group, *P* < 0.05

MDA and GSH, two oxidative stress indicators, were assessed in the stomach and liver of rats in this investigation. The ulcer and three protection groups had higher MDA concentrations. The ulcer group displayed the most significant levels of MDA in the liver and stomach. (Fig. 4a, b). Also, the GSH concentration was increased in the ulcer and protection groups in both the stomach and liver (Fig. 4c, d).

### Gene expression

**Fig. 5:**
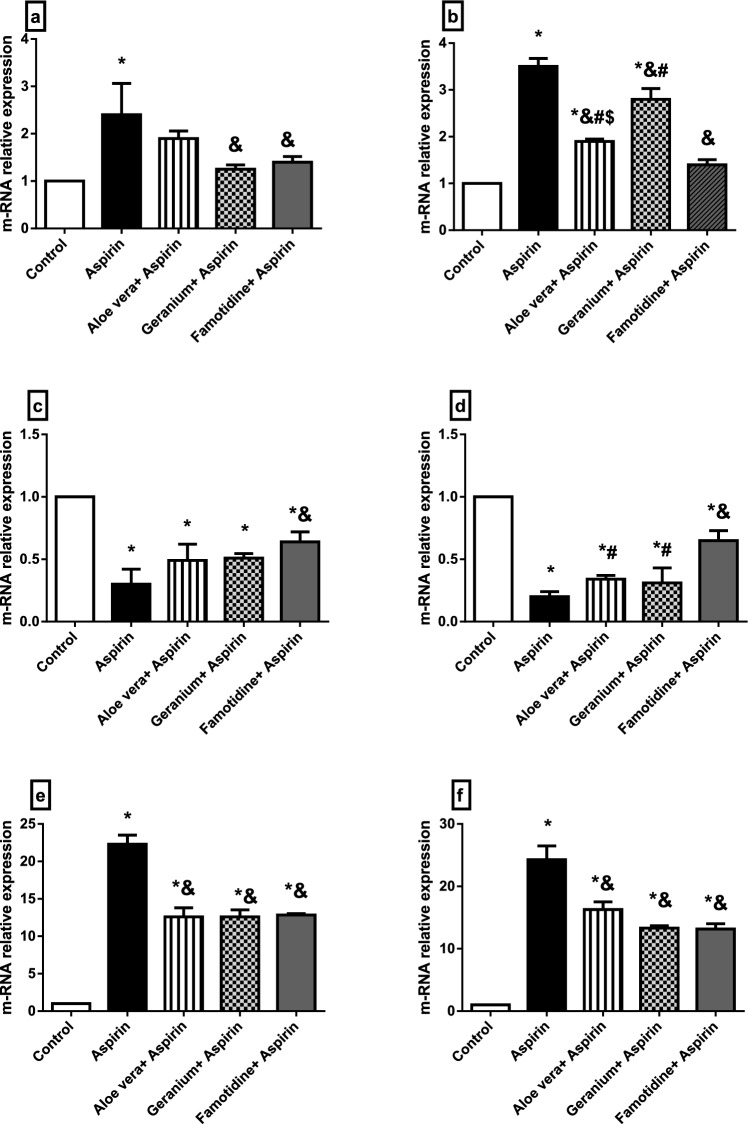
**a**–**f** The mRNA relative expression levels of oxidative stress-related genes in the stomach and liver of rats showing the transcript level of HO-1 gene in stomach **a** and liver **b;** the transcript level of keap-1 gene in stomach **c** and liver **d**; the transcript level of Nrf-2 gene in stomach **e** and liver **f**. Data are represented as mean ± SEM. * Significantly different from the control group, & significantly different from the Aspirin group, $ significantly different from the Geranium + Aspirin group, # significantly different from the Famotidine + Aspirin group, *P* < 0.05

**Fig. 6:**
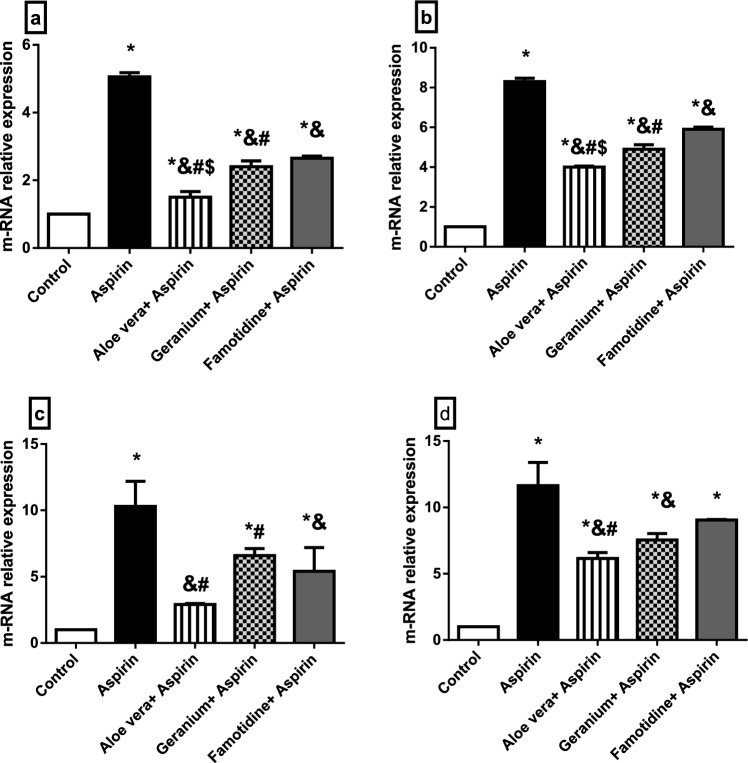
**a**–**d** The mRNA relative expression levels of inflammation-related genes in the stomach and liver of rats showing the transcript level of NF-kB gene in stomach **a** and liver **b**; the transcript level of COX-2 gene in stomach **c** and liver **d**. Data are represented as mean ± SEM. * Significantly different from the control group, & significantly different from the Aspirin group, $ significantly different from the Geranium +Aspirin group, # significantly different from the Famotidine + Aspirin group

qRT-PCR analysis for rat stomach and liver were used to determine the relative expression levels of a few genes linked to inflammation and oxidative damage. In the stomach and liver, the ulcer group had higher transcript levels of the oxidative stress-related genes Nrf-2 and HO-1 than the other groups (Fig. 5a, b, e, f). In contrast, the transcript level of the Keap-1 gene was down-regulated in the liver and stomach of the four groups compared to the control group. The ulcer group showed the most down-regulation, while the famotidine group showed the least down-regulation (Fig. 5c, d). In the liver and stomach, the transcript levels of the inflammatory-related genes NF-kB and COX-2 were considerably higher in the four groups than in the control group. The ulcer group showed the most significant up-regulation, whereas the *Aloe vera* group showed the least up-regulation (Fig. 6a–d).

### Histopathological and immunohistochemical findings

**Fig. 7:**
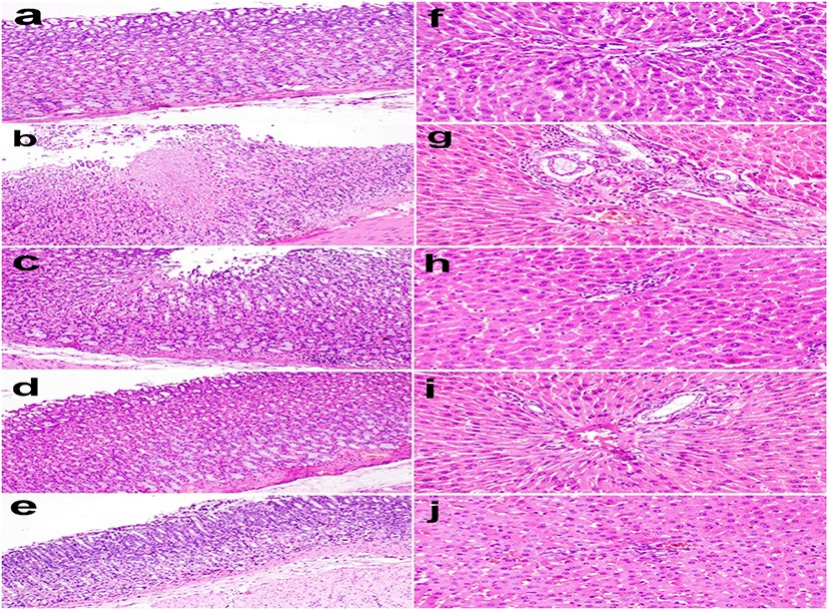
**a**–**e** Stomach of rat showing **a** normal histological structure of gastric mucosa in the control group; **b** severe focal cone-shaped necrotic foci in the positive control group; **c** mild focal detachment of lining epithelium in *Aloe vera* group; **d** mild histopathological alteration in the gastric mucosa in Geranium group; **e** Thinning of the gastric mucosa in Famotidine group; (Hematoxylin and Eosin stain × 200). **f**–**j** liver of rats showing **f** standard histological structure of the portal area in the control group; **g** Periportal leukocytes infiltration in the positive control group; (H and E stain × 400); **h** Few periportal leukocytes infiltration in *Aloe vera* group; **i** Normal histological structure in Geranium group; **j** Mild periportal leukocytes infiltration in Famotidine group (Hematoxylin and Eosin stain × 400)

Rats in the control group had intact mucosal epithelium and gastric glands, as seen by microscopic analysis of their stomach mucosa (Fig. 7a). The gastric mucosa microscopy in the positive control group revealed severe multifocal cone-shaped necrotic foci that extended from the depth of the stomach glands to the tunica musculosa, along with ulceration, thinning and detachment of the lining epithelium. Dysplastic alterations were seen in the glandular epithelium. Leukocytes, such as mononuclear cells and eosinophils, were invading the congested blood vessels of the interstitial tissue and submucosa (Fig. 7b). Rats in the *Aloe vera* group had a lessening of the lesions as seen by microscopy of their stomach in which the number and size of microscopic ulcers seen in the stomach mucosa were reduced. The tunica musculosa was not affected by the more superficial ulcers. Mild localised leukocyte infiltration and mononuclear cells were visible in the submucosa (Fig. 7c). Rats in the Geranium group had improved histoarchitecture of the gastric mucosa, as compared to the rats in the positive and *Aloe vera* groups, according to stomach microscopy. Leukocyte infiltration, stomach ulceration, and epithelial desquamation were less common in this group (Fig. 7d). The microscopy of the stomach of rats in the Famotidine group showed mitigation of the histopathological lesions observed in the positive control group’s. There were some submucosal leukocyte infiltration, desquamation of epithelial cells, dysplastic alterations in the glandular epithelium, and thinning of the stomach mucosa (Fig. 7e). The livers of the control group of rats underwent microscopic examination. The hepatic cords were oriented radially from the central vein to the portal region, a typical histological arrangement (Fig. 7f). Livers of the positive control group had moderate focal periportal leukocyte infiltration with eosinophils and mononuclear cells, as seen under the microscope. Hepatocytes in the liver displayed mild focal solitary necrosis, which was accompanied by enhanced cytoplasmic eosinophilia and pyknotic nuclei (Fig. 7g). Rats in the *Aloe vera* group had mild histopathological changes in their livers, including a few mononuclear cells in the periportal region and mild hepatocyte necroses, which were visible under the microscope. Binucleation might be seen in some hepatocytes (Fig. 7h). Mild histopathological changes were also visible in the rats' livers under the microscope in the geranium group (Fig. 7i). Rats in the Famotidine group had mild periportal leukocyte infiltration mild leukocytosis, and binucleation of hepatocytes, as seen under a microscope (Fig. 7j).

**Fig. 8:**
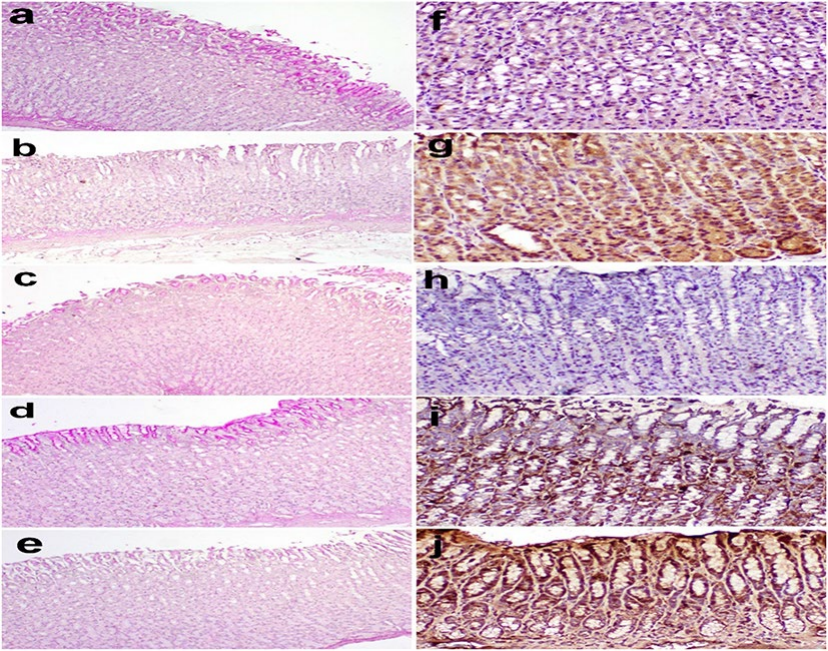
**a**–**e** Stomach of rat stained by PAS stain showing** a** strong positive PAS reaction in the mucus cells of the apical part of the stomach in the control group**; b** depleted reaction in the positive control group**; c** moderate reaction in the mucous cells of the apical part of the stomach in *Aloe vera,* pretreated group**; d** strong reaction in the mucous cells of the apical part of the stomach in Geranium group;** e** mild reaction in the stomach in Famotidine group (PAS stain × 200).** f**–**j** Immunohistochemistry of TNF-α cytokines in rat stomach showing** f** mild immunoreaction in epithelial cells in the control group; **g** strong reaction in epithelial cells in control positive group; **h** mild immunoreaction in epithelial cells in *Aloe vera* group;** i** moderate immunoreaction in some epithelial cells in Geranium group; **j** Strong reaction in epithelial cells in Famotidine group (Immuno-peroxidase × 400)

PAS stain indicates the number of mucous cells in the gastric mucosa. In the present study, microscopy of the stomach stained with PAS revealed a strong positive reaction in the mucus cells of the apical part of the stomach in the control group (Fig. 8a), a depleted reaction in the positive control group (Fig. 8b), a moderate reaction in the mucous cells of the apical part of the stomach in *Aloe vera* group (Fig. 8c), a strong reaction in the mucous cells of the apical part of the stomach in Geranium group (Fig. 8d), and mild PAS staining reaction in the stomach in Famotidine group (Fig. 8e). The immunohistochemistry of TNF-α in rat stomachs showed mild immunoreaction in epithelial cells in the control group (Fig. 8f), compared to a strong reaction in epithelial cells was detected in the positive control group (Fig. 8g). Mild immunoreaction in epithelial cells in *Aloe vera* group (Fig. 8h). Moderate immunoreaction in some epithelial cells in Geranium group (Fig. 8i), and strong reaction in epithelial cells in Famotidine group (Fig. 8j).

## Discussion

Gastric ulcer is one of the most upsetting health disorders seeking alternate therapy after the failure of most antiulcer drugs. Herbal medicines are currently in high demand for primary healthcare globally, not just because they are affordable but also for increased cultural acceptability, better compatibility with the human body, and minimal side effects. Our study reported that the pretreatment with *G. robertianum* L. leaves and *Aloe vera* gel powder displayed a significant gastroprotective effect besides antioxidant activity in Aspirin®-induced gastric ulcers. They were, moreover, restoring the stomach architecture and alleviation the Aspirin®-induced anxiety-like behaviour and motor deficits. All these beneficial effects could be attributed to the high polyphenol content of Geranium and *Aloe vera*, with their potent antioxidant activity (Amaral et al. 2009; Neagu et al. 2010). Antioxidants are essential for avoiding numerous oxidative stress-related disorders, such as cancer, heart disease, nerve deterioration, and gastroduodenal ulcers (Bhattacharyya et al. 2014; Chung et al. 2018).

Moreover, flavonoids in both herbs had gastroprotective and antiulcer effects, which play an essential role in strengthening the protective factors (mucus, bicarbonate, prostaglandins, and antioxidant enzymes) besides resisting the aggressive factors (gastric acid, pepsin, and oxidative stress) (Zhang et al. 2020). As shown, *G. robertianum* L. leaves and *Aloe vera* gel powder had significantly different antioxidant activity (*p*≤0.05). This effect could be attributed to the higher polyphenols content, which had antioxidant and anti-inflammatory properties (Girard et al. 2019), particularly the hydrolysable tannin (geraniin, vanillin, and catechin), flavonoids, and phenolic acids. Geraniin is the primary phenolic component among several Geranium species. Geraniin can reduce oxidative stress by restoring serum antioxidants, glutathione redox equilibrium, and oxidative stress indicators (Chung et al. 2018). Also, geraniin exhibited higher radical scavenging activity (RSA) than L-ascorbic acid and was effective at enhancing the activity of superoxide dismutase (SOD) (Thitilertdecha et al. 2020).

Additionally, vanillin and catechin are important phenolic compounds found in geranium. Vanillin decreased stomach levels of Thiobarbituric Acid Reactive Substances (TBARs), Lipid peroxidation byproducts and enhanced GSH and SOD, which affected lipid peroxide levels in gastric tissue, according to Katary and Salahuddin (2017). Using vanillin as a pretreatment can help reduce stomach acid output. The major phenolic acid in *G. robertianum* L. leaves was chlorogenic, the most efficient polyphenol, followed by gallic and Ellagic acid. Chlorogenic and gallic acids have been shown to have potent antiradical activities, and therefore they are most likely to contribute to the overall antioxidant activity demonstrated for geranium (Catarino et al. 2017). Also, Rutin, the primary flavonoid compound in geranium, followed by Naringenin, querectin, and Myricetin, can play a role in the antioxidant activity described by Ivancheva and Petrova (2000). Furthermore, several biological effects of flavonoids have also been connected to modulatory actions in the cell by affecting the cellular processes of signal transduction mediated by oxidants, which further control the inflammatory response (Rahman et al. 2006). Ginwala et al. (2019) also related the anti-inflammatory properties of flavonoids to their ability to reduce the production of reactive oxygen species (ROS) and downregulate several inflammatory mediators via essential signalling pathway inhibition.

*Aloe vera* has possessed antioxidant properties over the past many years. Our results revealed a high content of phenolic compounds in *Aloe vera,* particularly Dihydroxybenzoic acid, the major phenolic acid, followed by Coumaric acid and Ellagic acid. Also, it contains high flavonoid content, such as Naringenin. The high phenolic compounds in *Aloe vera* had antioxidant activity due to their redox properties (Muthukumaran et al. 2018). The High-performance liquid chromatography (HPLC) analytics of *Aloe vera* extracts indicated the presence of alkaloids, carbohydrates, tannin, steroids, and triterpenoids (Patel et al. 2012). Among these phytoconstituents, flavonoids have been demonstrated to protect against inflammation, allergies, platelet aggregation, free radicals, bacteria, viruses, hepatotoxins, tumours, and ulcers. Flavonoids also play a critical role in antioxidant activity (Eleazu et al. 2012). *Aloe vera* gel's antioxidant activity may be comparable to the quantity of ascorbic acid, which scavenges the free radicals that cause cellular damage, ageing, and cardiovascular illnesses (Kim et al. 2006). According to Riyanto and Wariyah (2012), *Aloe vera* extract has an antioxidant activity with a percentage of Radical Scavenging Activity (RSA) of 35.17%. Additional studies conducted in vitro and in vivo have demonstrated that *Aloe vera's* peptide and polypeptide fraction lowers inflammation by reducing inflammatory cytokines (Babu et al. 2019).

High gastric secretion volume and low pH of gastric juice produce significant gastric mucosal injury at the level of stomach architecture (Yi et al. 2015). After therapy, the *Aloe vera*-treated group's mean maximum length of stomach ulcers decreased compared to the ulcer group (Eamlamnam et al. 2006). Comparing the Geranium group to the positive and *Aloe vera* groups, the data showed an improvement in the histoarchitecture of the stomach mucosa. In a recent study, it was shown that the combination of polyphenolic compounds (flavonoids and phenolic acids) and tannins could treat stomach ulcers. Via several mechanisms, such as their capacity to scavenge free radicals and their antioxidant properties, they protect the gastric mucosa from ulcer-causing substances agents, enhance mucus production and antisecretory activity, and prevent the *Helicobacter pylori* development (Mota et al. 2009). As well flavonoids have been found to have antiulcer properties (Maury et al. 2012). Moreover, Al-Amin et al. (2012) demonstrated that the plant essential oils exhibit antiulcer action against Aspirin® plus, pylorus, ligation, and ethanol-induced rats and terpenes demonstrated substantial antiulcer efficacy in experimental animals' models against several ulcer-causing substances. The antiulcer and gastroprotective effects of Quercetin and its glucosides have also been discovered in numerous studies. Following pretreatment with vanillin, the indomethacin-induced histological changes, free, total acidity levels, ulcer index and gastric juice volume all decreased (Katary and Salahuddin 2017). Moreover, catechin showed a potent protective effect against stomach mucosal lesions and reduced the production of histamine, somatostatin, and gastrin. According to Sato et al. (2002), the nutritional constitution of *G. robertianum* L. has not received considerable attention. Several aqueous ethanolic extracts (50%, 70%, and 96% ethanol) from Romanian plant leaves were measured for their amounts of protein 1.117–2.242 g/mL and reducing sugars 257.2–479.5 g/mL (Neagu et al. 2010). Furthermore, reducing sugars, polysaccharides, proteins, and amino acids were present in the plant's aqueous extracts (Paun et al. 2011). *Aloe vera* gel is mainly composed of water (90%). In comparison, the remaining 1% solids materials consist of sugars (monosaccharides and polysaccharides), protein, lipids, vitamins A, C, and E, minerals (Zinc, Selenium), antioxidant enzymes (SOD and glutathione peroxidase enzymes) as well as phenolic compounds and other constituents (Boudreau and Beland 2006; Pandey and Singh 2016). Our presented data agreed with the results of Shubhra et al. (2014) and Hamidpour et al. (2017).

Behaviorally, there was evidence of anxiety and motor impairment associated with pain and inflammatory disorders of the GIT in the tested rat groups. Our findings showed that the pretreatment of anxious rats with Famotidine, *Aloe vera*, or Geranium completely alleviated Aspirin® anxiogenic effect*.* It supported Tabatabaei et al. (2017), who showed that medicinal plants like *Aloe vera* gel had a beneficial impact in lowering anxiety, motor impairment, or deficits. Moreover, increase locomotor and exploratory behaviour in rats. These results might be attributed to the antioxidant activity of *Aloe vera* in the hippocampus and cerebral cortex, which improves motor and cognitive behaviour (Parihar et al. 2004). Also, the anti-inflammatory potential of the polyphenolic compounds of *G. robertianum* L. aqueous extracts may alleviate anxiety (Catarino et al. 2017). Also, inhibiting gastric injury by *Aloe vera* or *geranium* may decrease anxiety-like behaviour (Werawatganon et al. 2014; Amaral et al. 2009). Our results agreed with Lo et al. (2020), who investigated that Geranium oil derived from geranium herbal medicine has anti-inflammatory and anxiolytic-like properties. Lim et al. (2014) demonstrated a relationship between mental traits and illnesses and gastric ulcer disease. Earlier research in animal models showed that GIT inflammation might be followed by behavioural changes, such as anxiety-like behaviour and changes in the biochemistry of the central nervous system (Bercik et al. 2010).

Furthermore, the EPM is an etiologically valid animal model of anxiety in which natural stimuli produce anxiety in rats, as fear of balancing on a relatively narrow raised platform (Dawson and Tricklebank 1995). In EPM, an anxiety index is calculated based on the frequency of entries into open arms (OAE) and the time spent there (Frih et al. 2012). Our findings were in line with those of Abd El-Ghffar et al. (2018), who demonstrated the anxiogenic effects of Aspirin® in mice using the OFT tests that displayed a shorter freezing period. Hamdan et al. (2020) demonstrated decreased crossing and rearing activities via ethanol-induced gastric ulcers in rats. Moreover, the previous study by Nku et al. (2015) showed a decrease in locomotor/exploratory and anxiety-like behaviour in Aspirin® treated mice compared to the control group. Gastric inflammation may alter brain function and affect animal anxiety-like behaviour by increasing proinflammatory cytokines such as (TNF-) (Luo et al. 2013) and through the upregulation of the transcript levels of oxidative stress (HO-1 and Nrf-2) and inflammatory (NF-kB and COX-2 genes) related genes as shown in our results. Treatment of the mental disorder may influence the course of gastric ulcer, apart from the direct benefits on mental health (Lim et al. 2014).

For understanding the essential protection mechanisms, tracing and determining oxidative stress biomarkers, MDA and GSH were analysed besides tracing the relative expression of principal proinflammatory, inflammatory, and oxidative stress-related genes. Moreover, histopathological and immunohistochemical examinations were conducted. Considering the oxidative stress biomarkers, unsaturated fatty acids generate MDA, an oxidative stress product, as a result of lipid peroxidation that is ROS-activated. MDA is, therefore, considered a lipid peroxidation biomarker that might be employed to detect and diagnose oxidative stress. The antioxidant GSH protects the stomach mucosa from the harmful effects of free radicals and peroxides. In order to lessen oxidative stress, it also works in conjunction with other antioxidant enzymes. As a result of Aspirin®-induced gastric ulcer, GSH concentration was increased. This rise may result from the body's antioxidant system responding to oxidative stress by scavenging it. The effects of *Acrostichum aureum* Linn. (WEAC) water extract against an ethanol-induced gastric ulcer in rats were examined. Rats exposed to ethanol showed an increase in MDA level and a decrease in GSH level (Wu et al. 2018).

The qRT-PCR analysis of some oxidative stress and inflammation-related genes in the stomach and liver of rats did not agree with the study of Badr et al. (2019), which showed a significant decrease in the Nrf-2 expression in adult male Wistar rats exposed to ethanol-induced gastric ulcer and pretreated with Raspberry Ketone (RK). In contrast, the Keap-1 gene showed down-regulation in the four groups than in the control group in both the liver and stomach. Basic studies have shown that catechin protects the gastric mucosa from ketoprofen-induced oxidative damage by up-regulating Nrf-2 and down-regulating Keap-1 (Yanaka 2018). Our results about Nrf-2 and HO-1 mRNAs agreed with Ueda et al. (2008), who showed the Nrf-2 nuclear translocation and the HO-1 up-regulation in the damaged stomach. The three types of protection had the same ameliorative effects on the stomach of rats in the case of oxidative stress-related gene expressions. Though, Famotidine was the best type of protection for the liver. Inflammation is an intricate immune system response to tissue damage defined by an increase in the production of proinflammatory cytokines from immune cells (Wu et al. 2018). The ameliorative effect of *Aloe vera* on the mRNA expressions of these inflammation-related genes in the liver and stomach was the best. NF-kB is a critical transcription factor that has long been thought to play a role in immunity and inflammatory responses. The NF-kB pathway has some inducible target genes. COX-2 gene is one of the inducible genes of the NF-kB pathway, which is induced and up-regulated after NF-kB stimulation (Natarajan et al. 2018; Wu et al. 2018). The ameliorative effect of *Aloe Vera* on the mRNA expressions of these inflammation-related genes in the liver and stomach was the best one.

These findings suggest that *Aloe Vera's* anti-inflammatory action, which is mediated via moderating the release of proinflammatory cytokines and activation of the NF-kB signalling pathway, is involved in treating stomach ulcers. The same as Wu et al. (2018) found that WEAC pretreatment dramatically reduced the levels of proinflammatory cytokines and blocked the activation of the NF-kB pathway by preventing IkB and p65 phosphorylation. Halter et al. (2001) suggested that COX-2 has a direct role in ulcer healing where COX-2 mRNA and protein expression is raised near the ulcer margin in a temporal and geographical relationship with increased epithelial cell proliferation and growth factor expression. These observations confirmed the role of COX-2 as a line of defence for the GIT mucosa and essential for maintaining mucosal integrity and ulcer healing.

Furthermore, the histopathological and immunohistochemical findings supported the previous results and agreed with Alazzouni et al. (2020). On the surfaces of endothelial cells, Aspirin® may stimulate the expression of cell adhesion molecules, damage to the stomach mucosa and the recruitment of leukocytes to inflammatory areas. Leukocyte adhesion has been demonstrated to impair microcirculation, causing ischemia and mucosal injury that form ulcers (Eamlamnam et al. 2006). Due to the acidic environment, a small dose of Aspirin® causes GI mucosal and systemic effects via inhibiting COX-1; In the absence of natural prostaglandins that encourage the creation of bicarbonate and mucus, Aspirin® remains nonionised, causing it to accumulate in stomach mucosal cells, changing their permeability, and leading to ulceration within minutes of administration (Cryer and Mahaffey 2014). *Aloe vera* reduces inflammation by acting as a bradykininase inhibitor and enhancing vascular permeability in the stomach's microscopy in the *Aloe vera* group. So, after a stomach ulcer develops, *Aloe vera*'s effects could reduce the influence of the inflammatory process, as well as the effects and leukocyte adherence. In the microscopy of the liver of rats, like our findings**,** exposure to Aspirin® was reported before to cause liver injury and increase the expression of caspase-3 (Mossa et al. 2014). From our data, the geranium and *Aloe vera* protected the stomach and liver against injury due to their anti-inflammatory and antioxidant potential. They both decreased TNF-α expression in the gastric mucosa. Proinflammatory cytokines like TNF-α are critical in developing gastric ulcers (Du et al. 2013). Besides, the increased TNF-α expression in gastric mucosa contributes further to gastric mucosa damage (Harakeh et al. 2022). Furthermore, *Aloe vera* can subside the inflammatory process by acting as a bradykinin inhibitor (Eamlamnam et al. 2006). Also, vanillin in geranium decreased the expression and activity of gastric NF-KB-p65, TNF-α, IL-1, IL-6 and IL-10 in the stomach (Ciciliato et al. 2022).

## Conclusion

The current study assessed the potential gastroprotection effect of *G. robertianum* L. leaves and *Aloe vera* gel powder against Aspirin®-induced gastric ulcers in rats by determining the essential protection mechanisms. They both displayed significant gastroprotective effects, alleviated the Aspirin®-induced anxiety-like behaviour and motor deficits by decreasing the gastric mucosal injury, and improved the stomach architecture by reducing TNF-α-expression in gastric mucosa, inflammatory, and oxidative stress-related genes (NF-KB, HO-1, Nrf-2) while increasing the Keap-1 gene. Based on the findings of this study, *G. robertianum* L. and *Aloe vera* gel might be used as a therapeutic medication or dietary supplement for treatment or preventing Aspirin®-induced gastric ulcers.

## Data Availability

All data generated or analysed during this study are included in this article.

## Abbreviations

COX-2: Cyclooxygenase-2
CRF: Corticotropin-releasing factor
CURP: Cairo University Research Park
DAB: Diaminobenzidine
DPPH: 1,1-Diphenyl-2-picrylhydrazyl
EPM: Elevated plus maze
FRAP: Ferric reducing power
*G. robertianum*: *Geranium robertianum*
GIT: Gastrointestinal tract
GSH: Reduced glutathione
GU: Gastric ulcers
HPLC: High-performance liquid chromatography
IL-10: Interleukin-10
IL-6: Interleukin-6
LC/ESI-MS/MS: Liquid chromatography/electrospray ionization-tandem-mass spectrometry
MDA: Malondialdehyde
NSAIDs: Nonsteroidal anti-inflammatory drugs
OAE: Open arms entries
OFT: Open-field test
PAS: Periodic acid–Schiff stain
PVN: Paraventricular nucleus
qRT-PCR: Quantitative real-time polymerase chain reaction
RK: Raspberry ketone
ROS: Reactive oxygen species
RSA: Radical scavenging activity
TBARs: Thio-barbituric acid reactive substances
TNF-α: Tumour necrosis factor-alpha
TPTZ: 2,4,6-Tripyridyl-*S*-triazine
UA: Ulcer area
UI: Ulcer index
VET-IACUC: Veterinary Institutional Animal Care and Use Committee
WEAC: Water extract of *Acrostichum aureum* Linn.
WHO: World Health Organization
